# Adaptation to heat stress by diversification of the vertebrate heat shock transcription factor family

**DOI:** 10.1098/rstb.2025.0274

**Published:** 2026-10-01

**Authors:** Ryosuke Takii, Mitsuaki Fujimoto, Akira Nakai

**Affiliations:** Department of Biochemistry and Molecular Biology, Yamaguchi University School of Medicine, Ube, Yamaguchi Prefecture, Japan

**Keywords:** heat shock, HSF, heat resistance, body temperature, vertebrate, evolution

## Abstract

Cells survive and reproduce by coping with acute or chronic exposure to a fixed elevated temperature or fluctuating temperatures. These properties are one of the main evolutionary forces and involve the heat shock response. This response is characterized by the induction of heat-shock proteins (HSPs) and is mainly regulated by heat-shock transcription factors (HSFs) in vertebrates. A single HSF is present in yeast and invertebrates, such as *Caenorhabditis elegans* and *Drosophila*, whereas vertebrates have evolved multiple HSFs. The potential of HSFs to induce *HSP* expression is linked to the amino acid conservation of their domains and regions that are responsible for transcriptional activation and appears to be associated with the homeothermic capacity of vertebrate animals in a thermally fluctuating environment. HSFs protect cells and organisms from heat stress by regulating not only their expression of HSPs that assist in protein folding and suppress protein misfolding and aggregation, but also the expression of genes related to protein clearance, metabolism, the cell cycle, DNA damage response, apoptosis, senescence, the cytoskeleton, extracellular matrix and inflammation. The diversification of *HSF* genes may expand the cellular capacity to adapt to thermal changes in vertebrate animals.

This article is part of the Theo Murphy meeting issue ‘ProteostaSys: a systems view of proteostasis’.

## 1. Introduction

All organisms live within a limited body temperature range owing to the optimal coordination of molecular, cellular and systemic processes [1]. Body temperature is affected by behaviours and environmental factors and exerts pleiotropic effects that have an impact on these processes. There are two primary responses of body temperature to fluctuating environmental temperatures: ectothermy and endothermy. Vertebrate ectotherms, including fishes, amphibians and reptiles, depend mainly on external heat sources, and their body temperature changes with ambient temperature. By contrast, endotherms, including aves (birds) and mammals, use internally generated heat to maintain body temperature, and their core body temperature is generally maintained at the appropriate level [2,3]. However, some endotherms with low body temperatures, such as monotremes, possess a low capacity to generate heat and are regarded as protoendotherms [4]. Moreover, the body temperature of endotherms is not completely uniform; it is nearly constant at the core but may markedly change in the extremities such as the skin, the temperature of which varies with ambient temperature [2]. Climate warming or exposure to high temperatures has detrimental effects on various molecular, cellular and systemic processes in all these vertebrates and may drive phenotypic and genetic diversification over evolutionary and ecological timescales [5,6]. It is also associated with pathological conditions such as accelerated ageing [7,8] and cardiovascular disease [9].

The heat-shock response (HSR) is a universal mechanism of cellular adaptation to high temperatures. It is characterized by the induction of a set of heat-shock proteins (HSPs) or chaperones that facilitate protein folding and the assembly of newly synthesized proteins [10]. They also prevent protein denaturation and the formation of aggregates and amyloid fibrils under proteotoxic stresses, including heat shock [11]. Therefore, HSR has been considered to protect cells and organisms from heat stress; however, there are many contradictions, which may be attributed to the complex physiological effects of heat [12]. In eukaryotes, the expression of HSPs is primarily regulated at the transcriptional level by evolutionarily conserved heat-shock transcription factors (HSFs) that bind to heat-shock response elements (HSEs), which are composed of inverted repeats of the consensus pentanucleotide nGAAn (n denotes any nucleotide) [13]. A single HSF exists in yeast, worms and fruit flies, whereas four HSFs (HSF1–HSF4) have been identified and characterized in vertebrates [14,15]. The HSF family members described to date remain as monomers, dimers or trimers in cells under unstressed or stressed conditions, and the trimeric form of HSFs may bind to the HSEs with high affinity. In accordance with this property, HSFs universally possess the oligomerization domain, or hydrophobic heptad repeat (HR)-A/B [16,17], as well as the DNA-binding domain (DBD) consisting of a winged helix-turn-helix motif [18–21]. After the independent isolation of budding yeast and Drosophila *HSF* genes [22–24], HSF1 and HSF2 cDNA clones were subsequently isolated from human and mouse libraries [25–27]. HSF1 was subsequently revealed to be the primary factor undergoing trimerization, the acquisition of HSE-binding activity and nuclear translocation in mammalian cells during heat shock [28,29]. Many researchers have since been focusing on the roles and regulatory mechanisms of HSF1 in a number of cellular processes, including the HSR, under physiological and pathological conditions [30–32]. HSF3 and HSF4 cDNA clones have been isolated from chicken and human libraries, respectively [33,34]. HSF3 was revealed be a pivotal regulator of the HSR in chicken cells, although it has long been regarded as an avian-specific factor [15].

The whole-genome sequences of a number of vertebrate species were recently elucidated and released to the public. With the development of these genome databases, the four *HSF* genes, including *HSF3*, have been identified in most vertebrate species with some notable exceptions. Experimental evidence has suggested that homeothermic capacity is associated with the functional diversification of *HSF* genes during vertebrate evolution. We herein summarize the conservation and diversification of functional domains and regions in vertebrate HSF family members, with an emphasis on HSF1 and HSF3, which are the primary factors responding to heat shock. The relationship between the homeothermic capacity of representative vertebrate species and the potential of HSFs to induce HSPs are then discussed. We also introduce a wide range of pathways regulated by HSF family members and discuss potential mechanisms underlying the acquisition of heat resistance.

## 2. Conservation and diversification of the amino acid sequences of vertebrate HSFs

To identify similarities among the HSF1, HSF2, HSF3 and HSF4 proteins from diverse vertebrate species, the putative amino acid sequences of HSFs from representative mammals, birds, reptiles and amphibians were used to generate a phylogenetic tree (supplementary material, figure S1A). HSF-related gene products (human HSF5, HSFX1 and HSFY1), belonging to the HSF superfamily, were also included as the outgroup [15]. HSF1 and HSF2 gene products were identified in all vertebrate species listed (supplementary material, figure S2). *HSF3*, located between *VSIG4* and *HEPH* on chromosome X, is a pseudogene in humans, and HSF3 gene products have not been identified in the corresponding syntenic regions in primates, including chimpanzee and gorilla. By contrast, HSF3 gene products have been identified in all other species. HSF4 gene products are present in most vertebrate species, except for monotremes (echidna and platypus) and turtles. The phylogenetic tree confirmed the clear separation of the HSF1 and HSF4 clusters from the HSF2 or HSF3 cluster (supplementary material, figure S1A). Comparisons of sequence similarities among the clusters showed that HSF1 is the most highly conserved factor in vertebrates (figure 1A). HSF2 is also a well-conserved factor, although HSF2 sequences are less closely related among mammalian subclasses (placentals, marsupials and monotremes) (figure 1B). By stark contrast, HSF3 is the most distantly related factor among vertebrate species (figure 1C). Similarly, HSF4 is also a distantly related factor among vertebrate species (figure 1D). Therefore, the *HSF3* and *HSF4* genes diverged more quickly than the *HSF1* and *HSF2* genes during vertebrate evolution. It is important to note that HSF4 proteins in placentals (human, mouse, guinea pig, squirrel and armadillo) are very well conserved.

**Figure 1.**
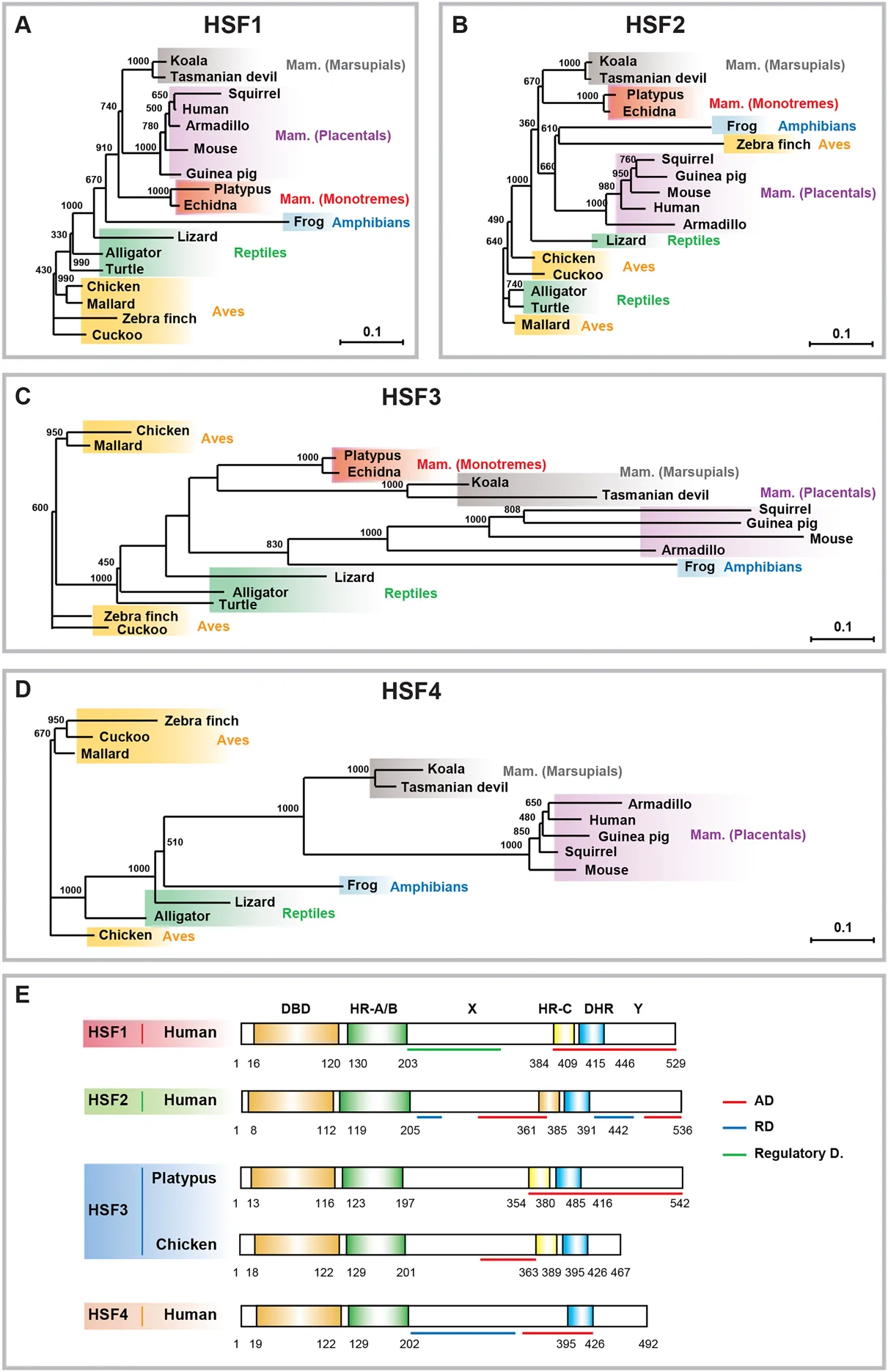
Phylogenetic trees of vertebrate HSF family members. (A–D) Unrooted trees were drawn using the NJ/UPGMA method implemented in MAFFT v. 7 [35] for HSF1, HSF2, HSF3 or HSF4 orthologues in vertebrates (except fishes). Numerals represent bootstrap values (1000 bootstrap replicates were performed). The bars represent 0.1 substitutions per site. HSFs in different classes and mammalian (Mam.) subclasses are indicated in different colours. The amino acid sequences used to construct the trees are shown in figure S1 in the supplementary material. (E) Diagrammatic representation of human HSF1, HSF2 and HSF4. and of HSF3 from platypus and chicken. The numbers of amino acids of each HSF are shown. DBD, DNA-binding domain; HR, hydrophobic heptad repeat; DHR, downstream of HR-C; region X, a region between the HR-A/B and HR-C; region Y, a C-terminal region downstream of the DHR [15]. Transcriptional activation domains (ADs) and repression domains (RDs) are indicated as red and blue bars, respectively. The regulatory domain (regulatory D.) in the X region in human HSF1 is indicated as a green bar. The AD of chicken HSF3 is markedly different from that of platypus HSF3 (supplementary material, figure S2).

We then examined the conservation of the amino acid sequences of HSFs that are responsible for transcriptional activation and repression, as well as those of the DBD and HR-A/B domains (figure 1E). HSF1 is a master regulator of HSR in mammalian cells (table 1), and, thus, the activation domain (AD) and activating and repressing modification (phosphorylation, acetylation and ubiquitylation) sites in human HSF1 have been intensively investigated and identified [32,38]. In addition to the highly conserved DBD and HR-A/B domains, the ADs containing the HR-C, DHR (downstream of HR-C) and Y region are well-conserved regions in all vertebrate species [39–41] (supplementary material, figure S2). The HR-C is known to directly inhibit the oligomerization of the HR-A/B and keeps HSF1 as a monomer under unstressed conditions [17,41]. The DHR, composed of a variant of the hydrophobic heptad repeat [34], is the alternatively spliced-out region containing the active phosphorylation site S419 [42,43]. The X region containing the regulatory domain, which represses the activity of AD under unstressed conditions and renders it inducible by heat shock [44], is also well conserved. Furthermore, activating (T120, S121, T142, S230, S326, S419 and K298) [45–51] and repressing (S303, S307 and K80) [52–55] modification sites are identical in all vertebrate species with only one or two exceptions (supplementary material, figure S3A). For example, the active phosphorylation site S326 is deleted or substituted in chicken and cuckoo. Consistent with its absence, the transcriptional activity of chicken HSF1 is low [56,57]. The other activating phosphorylation sites, S320 and S529, are only conserved in non-monotreme mammals and placentals, respectively [58,59].

**Table 1.** Thermoregulatory properties of vertebrate species and the ability of their HSF1 and HSF3 proteins to induce HSP70.

classification			HSP70 induction^a^		characteristic		
class	subclass	species	HSF1	HSF3	body temp. (°C)	ref.^e^	thermoregulation
	plasentals	human (*Homo sapiens*)	++^b^	(pseudo)^d^	36.1–37.2	212ˏ213	
		mouse (*Mus musculus*)	++^b^	–^b^	36.0–37.0	214	
		guinea pig (*Cavia porcellus*)	n. t. (++)^c^	n. t. (–)^c^	38.7–39.1	215	
		squirrel (*Ictidomys tridecemlineatus*)	n. t. (++)^c^	n. t. (–)^c^	34.0–39.0	216ˏ217	endotherm
mammals		armadillo (*Dasypus novemcinctus*)	n. t. (++)^c^	n. t. (–)^c^	34.0–36.0	218	
	marsupials	tasmanian devil (*Sarcophilus harrisii*)	n. t. (++)^c^	n. t. (++)^c^	31.3–38.6	219	
		koala (*Phascolarctos cinereus*)	n. t. (++)^c^	n. t. (++)^c^	35.4–36.9	220	
	monotremes	echidna (*Tachyglossus aculeatus*)	++	++	28.0–32.0	221	protoendotherm^f^
		platypus (*Ornithorhynchus anatinus*)	++	++	25.0–33.0	222	
		cuckoo (*Cuculus canorus*)	n. t. (+)^c^	n. t. (++)^c^	25.0–33.0	223	
aves		zebra finch (*Taeniopygia guttata*)	+	++	36.5–39.5	224	endotherm
		mallard (*Anas platyrhynchos*)	++	–	40.5–41.5	225	
		chicken (*Gallus gallus*)	±^b^	++^b^	40.5–42.5	226	
		turtle (*Mauremys reevesii*)	n. t. (++)^c^	n. t. (++)^c^	<33.0	227	
reptiles		alligator (*Alligator mississippiensis*)	n. t. (++)^c^	n. t. (++)^c^	<33.5	228	ectotherm
		lizard (*Anolis sagrei*)	++^b^	++^b^	<40.0	229	
amphibians		frog (*Xenopus tropicalis*)	++	++	<36.5	230	ectotherm

a Ability to induce HSP70 upon heat shock when each HSF was overexpressed in HSF1-null MEF cells. The level of the HSP70 protein in heat-shocked HSF1-null MEF cells expressing each HSF relative to that in heat-shocked wild-type cells. Marks indicate: ++, >70%; +, <70%; ±, <10%; −, no induction [36].

b The ability to induce HSP70 has been confirmed by gene knock-out or knock-down in cells from each species.

c Prediction of the ability of HSF1 and HSF3 proteins to induce HSP70 through their identities of the C-terminal activation domains and modification sites (supplementary material, figure S3). n. t., not tested.

d The human *HSF3* is a pseudogene [37].

e References for body temperatures in vertebrates are listed in supplementary material.

f Protoendotherms are organisms that display a primitive or inadequate thermoregulatory ability [4].

HSF3 is another factor that may induce the expression of HSPs by itself under heat shock (table 1). It is a dominant regulator of HSR in chicken cells [60], and it cooperates with HSF1 to maximally induce *HSP* expression in lizard cells [61]. On the other hand, HSF3 lacks the ability to induce *HSP* expression and is dispensable for HSR in mouse cells [37]. These findings suggest marked changes in the amino acids of HSF3 proteins that are responsible for transcriptional activation. The DBD and HR-A/B of HSF3 proteins are highly conserved in all vertebrates listed, and the HR-C of HSF3 proteins is well conserved in vertebrates, except for mammalian placentals (supplementary material, figure S2). HSF3 proteins in placentals possess the HR-C-like domains, in which hydrophobic amino acids are not well conserved [37]. The AD of platypus HSF3 contains the HR-C, DHR and Y region, like that of human HSF1 discussed above [36]. The C-terminal Y region of HSF3 proteins is not conserved, and the DHR is absent in mammalian placentals. By contrast, the Y region is well conserved in mammalian marsupials and monotremes, birds and reptiles, except for chicken, lizard and frog (supplementary material, figure S2). The C-terminal end (approx. 30 amino acids) is a highly conserved region in most vertebrates, except for chicken, lizard and frog. The Y regions in chicken and lizard are very short, and the AD in chicken is located upstream of the HR-C (within the X region) [62]. A part of this AD is well conserved in many species, including lizard and frog (supplementary material, figure S3B). Since HSF3 proteins from chicken, lizard and frog possess the potential to induce the expression of HSPs upon heat shock [61], the region corresponding to the chicken AD in lizard and frog may also be responsible for their induction.

HSF2 and HSF4 cannot induce the expression of HSPs by themselves upon heat shock [63,64], although they modulate HSF1-mediated *HSP* expression [65,66]. Furthermore, HSF2 plays important roles in neurogenesis and spermatogenesis in cooperation with HSF1 [67–69], and HSF4 is required for the development and maintenance of mouse and human lenses [70–72]. Human HSF2 possesses several activation and repression domains in the X and Y regions [73], while human HSF4 possesses an AD in the DHR and X region [34]. The DBD, HR-A/B and HR-C of HSF2 proteins are highly conserved domains in all vertebrate species listed, and the amino acid sequences of the DHR and X and Y regions are well conserved (supplementary material, figure S2). Therefore, these domains and regions in vertebrate HSF2 proteins appear to be indispensable. The DBD, HR-A/B, DHR and X and Y regions of HSF4 proteins are highly conserved domains or regions in mammalian placentals, similar to those in HSF2 proteins. In addition, the DBD and HR-A/B of HSF4 proteins are two well-conserved domains in all vertebrate species. However, the amino acid sequences of the Y regions of HSF4 proteins in birds, reptiles and amphibians are not conserved with those in mammalian placentals (supplementary material, figure S2). It is possible that HSF4 may not play the same role among vertebrate species.

In vertebrate genome evolution, it has been hypothesized that a first whole-genome duplication (WGD) event occurred after the divergence of invertebrates and before jawless vertebrates, followed by a second WGD event (more than 440 million years ago (Mya)) [74–76]. The four *HSF* genes may have been generated through the two rounds of WGD in vertebrates [15,61]. A third WGD event, which is specific to fish, occurred in the common ancestor of all teleost fish (approx. 300 Mya) [77,78], and most of the duplicated genes became non-functional through the accumulation of deletions and mutations [79]. HSF1, HSF2 and HSF4 gene products, but not HSF3 gene products, have been identified in representative teleost fishes, including zebrafish and rainbow trout [80–82]. Since the putative amino acid sequences of these fish HSFs are very distantly related to those from the other vertebrates, they were separately used to generate a phylogenetic tree (supplementary material, figure S1B). In fishes, the HSF2 and HSF4 clusters are closely related to each other, and are distantly related to the HSF1 cluster. It is important to note that there are two distinct *HSF1* and *HSF2* genes in salmonids such as rainbow trout and king salmon (supplementary material, figure S4), which may be owing to the salmonid-specific fourth WGD event that occurred recently (approximately 100 Mya) [83]. Nevertheless, only a single *HSF4* gene has been identified in king salmon. Furthermore, the C-terminal Y regions in HSF4 are very short in most fishes. These findings suggest the fish-specific evolution of *HSF* genes among vertebrate species.

## 3. Homeothermic capacity and the potential of HSFs to induce the HSR

All vertebrate animals inhabit appropriate temperature environments. The body temperature of vertebrate ectotherms changes with ambient temperature fluctuations, and body temperature is one of the most important factors affecting the physiological performance and behaviour of vertebrate ectotherms (figure 2A). Vertebrate endotherms maintain a relatively constant core body temperature regardless of the external environment, but they also experience regional and temporal variations in their body temperature. Therefore, adaptive mechanisms for high-temperature environments may have evolved differently across vertebrate species with varying homeothermic capacities, that is, the ability to maintain a stable internal body temperature despite changes in the external environment [86,87]. It is also possible that tissue-specific adaptive mechanisms have evolved.

**Figure 2.**
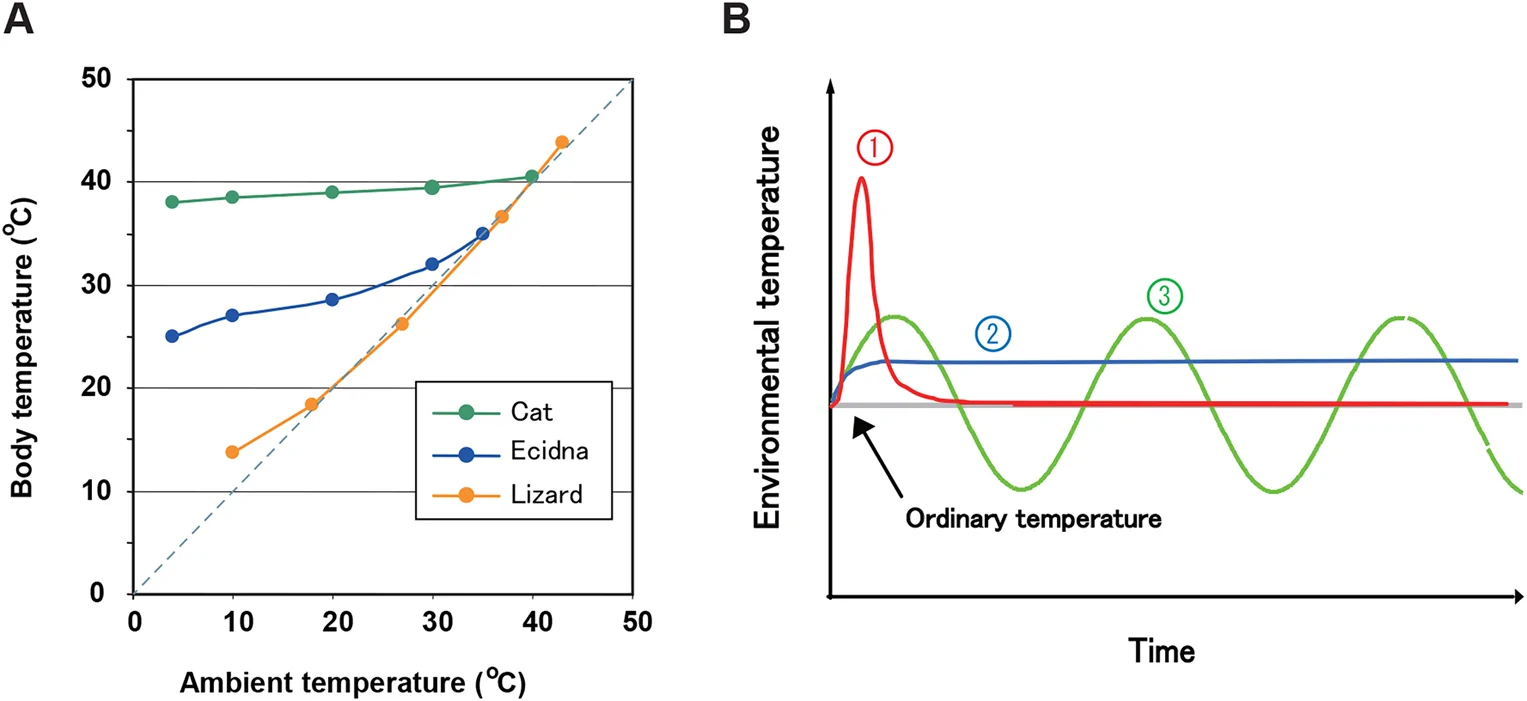
Body temperature and environmental temperature. (A) Body temperature responses of an endotherm (cat), a protoendotherm (echidna, *Tachyglossus aculeatus*) and an ectotherm (lizard, *Ctenophorus nuchalis*) to changing environmental temperatures [2,84,85]. (B) Different types of elevated environmental temperatures. Acute (1) and chronic (2) exposures to a fixed elevated temperature and exposure to a thermally fluctuating environment (3) may exert different effects on the physiological performance and behaviour of vertebrate animals and the physiology and survival of these cells.

As described above, HSF1 acquires HSE-binding activity in human and mouse cells during heat shock [28,29], and the disruption of the *HSF1* gene resulted in a lack of the heat shock-induced *HSP* expression in mouse embryonic fibroblasts (MEFs) [88,89]. In chicken cells, HSF1 and HSF3 both acquired HSE-binding activity upon heat shock [90], and the *HSF3* gene disruption resulted in a severe reduction in the induction of HSPs in chicken DT40 cells [91]. By contrast, the disruption of the chicken *HSF1* gene or mouse *HSF3* gene did not affect the induction of major HSPs [37,56]. These findings raised the question of whether HSF3 or HSF1 is involved in the HSR in chicken, birds or a variety of vertebrate species. An analysis of the *HSF1* and *HSF3* genes from other representative vertebrate species has advanced following the availability of their complete whole-genome sequences (table 1). To examine the potential of each HSF to induce HSPs, it is overexpressed in HSF1-null MEF cells, and the expression of endogenous HSP70 is examined during heat shock. It is already known that the expression of HSP70 was strongly induced in HSF1-null MEF cells overexpressing not only human HSF1 and chicken HSF3, but also *Drosophila* HSF [91].

The cDNA clones of four HSFs were subsequently isolated from the ectotherm, the brown anole lizard (*Anolis sagrei*), based on its sequence data [61,92]. The findings obtained revealed the potential of brown anole HSF1 and HSF3, but not HSF2 or HSF4, to maximally induce HSP70 (table 1). HSF1 and HSF3 in the brain and heart of brown anole both acquired HSE-binding activity upon heat shock, similar to chicken cells. A detailed analysis conducted using a lizard GL-1 cell line derived from the tokay gecko (*Gekko gecko*) showed that HSF1 and HSF3 exist as a monomer and a dimer, respectively, and are converted to a trimer that binds to the HSE during heat shock. Furthermore, HSF1 and HSF3 were found to cooperate in order to maximally induce the expression of HSPs [61]. Similarly, HSF1 and HSF3 from another ectotherm, the western clawed frog (*Xenopus tropicalis*), have the potential to maximally induce HSP70 (table 1). HSF1 and HSF3 both acquire HSE-binding activity upon heat shock in tissues and cells from the western clawed frog, which is associated with a transition from a monomer or dimer to a trimer [61].

Monotremes, including platypus and echidna, are egg-laying mammals that are characterized by a low body temperature (approx. 30°C) and metabolic rate and, thus, are considered to be protoendotherms [4]. Owing to their low capacity to generate heat, they become hypothermic under cold ambient temperatures [93,94]. To investigate whether HSF1 and HSF3 from monotremes induce the expression of HSPs, their cDNA clones were isolated and characterized [36,95]. The findings obtained showed the potential of HSF1 and HSF3 from both platypus and echidna to maximally induce HSP70 (table 1). Moreover, platypus HSF3 remains as a dimer and is converted to a trimer during heat shock when it is expressed in human fibroblasts (R. Takii & A. Nakai 2026, unpublished data). These findings strongly suggest that HSF3 and HSF1 regulate the HSR in vertebrate animals with a low body temperature and metabolic rate to cope with acute elevations in body temperature in the wild (see §4 below). The mean body temperature of two marsupials, Tasmanian devil and koala, is almost the same as that of humans (approx. 36.5°C); however, their daily body temperature amplitude is higher, with the lowest body temperature of approximately 31 or 32°C, at least owing to a low metabolic rate [96–98] (table 1). Furthermore, the putative amino acids of HSF3 proteins from Tasmanian devil and koala are well conserved with those from platypus HSF3 (supplementary material, figure S2), suggesting their potential to induce HSP70 (table 1).

There is still a missing link in the evolution of *HSF* genes in endotherms, including birds, which exhibit significant daily fluctuations in body temperature caused by high behavioural activity requirements (e.g. flying) [99]. An analysis of HSF1 and HSF3 from mallard and zebra finch, which are distantly related to chicken among all bird species [100], revealed that mallard HSF1 possesses the potential to maximally induce HSP70, whereas the potential of zebra finch HSF1 is weak. On the other hand, zebra finch HSF3, but not mallard HSF3, has the potential to maximally induce HSP70 [36]. Taken together with findings on human, mouse and chicken HSFs, at least one of the HSF1 and HSF3 proteins may possess the potential to induce the maximal HSP70 expression in birds and placental mammals with a high body temperature and metabolic rate. Overall, the potential of vertebrate HSF1 and HSF3 to induce the HSR is closely related to their homeothermic capacity.

## 4. Mechanisms of the acquisition of heat resistance

### (a) All HSFs play roles in adaptation to high temperatures

It has now been proven that HSPs protect critical cellular structures and essential functions from the deleterious effects of heat shock [12,101,102]. Classical experiments have shown that cells pre-exposed to moderately elevated temperatures acquire resistance to more extreme, short-term lethal temperatures [103]. The survival of these cells is assessed by their proliferative capacity measured according to their colony-forming ability, and this phenomenon is known as thermotolerance. The acquisition of thermotolerance correlates with the synthesis of HSPs under some heat shock conditions [104–106], but not under other conditions [107–109]. This discrepancy may stem from the complex effects of heat shock. Many studies have examined the effects of acute and chronic exposure to a fixed elevated temperature (extreme or moderate) on the physiology and survival of cells (figure 2B). It is also important to understand the effects of fluctuating temperatures because most organisms live in thermally fluctuating environments [110,111], and temperature fluctuations are theoretically predicted to exert different effects on organisms from stable elevated temperatures [112,113]. Daily temperature fluctuations have been shown to increase the rates of development and survival in insects at cold mean temperatures, but such fluctuations decrease these rates at warm mean temperatures [114,115], and they markedly affect the performance of vertebrates, particularly ectotherms [116–118].

Since mouse HSF1 is responsible for the induced expression of HSPs, HSF1 was found to protect MEF cells pretreated with moderate heat shock (e.g. 43°C for 20 min) from apoptosis caused by exposure to extreme heat shock (e.g. 45°C for 30 min), and its deficiency abolished thermotolerance [88,89]. In chicken B lymphocyte DT40 cells, HSF3 deficiency resulted in a severe reduction in the induced expression of HSPs upon heat shock and thermotolerance (e.g. 46°C for 60 min after a pretreatment at 43°C for 30 min) [60]. Chicken HSF1 has a limited ability to induce the expression of HSPs, and, thus, HSF1 deficiency does not affect the acquisition of thermotolerance. HSF1-deficient DT40 cells were shown to be highly sensitive to chronic high temperatures (e.g. 43.5°C for 24 h) and while a deficiency in both HSF1 and HSF3 caused cell death, this deficiency can be fully restored by the overexpression of human HSF1, but not chicken HSF3 [91]. These findings suggest a role for non-HSP pathways regulated by human and chicken HSF1 in adapting to chronic exposure to a constant elevated temperature. Alternatively, given the higher threshold temperature for HSF3 activation [62], HSF3 may undergo earlier attenuation under chronic heat stress. Furthermore, the role of HSF1 in the adaptation of human fibroblasts to a daily temperature fluctuation (e.g. 39.5°C for 10 h and 30°C for 14 h each day for 14 days) was investigated [36]. HSF1 deficiency resulted in a marked reduction in cell proliferation and survival under daily temperature fluctuations, and these decreases were restored by the overexpression of HSF1 proteins from human, chicken, mallard and platypus, but not by that of HSF3 proteins from chicken, zebra finch, platypus and echidna. Therefore, vertebrate HSF1 proteins play roles in adaptation to not only acute exposure to an extreme temperature (thermotolerance), but also to chronic exposure to a constant elevated temperature and a fluctuating temperature (figure 2B). It is important to note that mouse HSF3 cannot induce HSP expression, whereas a reduction in HSF3 increases the sensitivity of MEF cells to chronic exposure to an elevated temperature (e.g. 42°C for 6 h) [37].

HSF2 does not induce the expression of HSPs by itself, but contributes to heat resistance. HSF2 accounts for part of the HSE-binding activity in mouse and human cells during heat shock (e.g. 42°C for 1 h or 43°C for 0.5 h) [28,119], and translocates to the nucleus and nuclear stress granules in human cells [120,121]. HSF2 forms a heterotrimer with HSF1 and positively or negatively contributes to the expression of HSPs in MEF cells during heat shock [65,122]. Indeed, HSF2 and HSF1 bind to almost the same promoters and enhancers of many target genes, including the *HSP* genes in mouse and human cells, before and after heat shock [123,124]. In contrast to HSF1 deficiency, HSF2 deficiency in MEF cells did not affect thermotolerance or cell survival during chronic exposure to a high temperature (e.g. 42°C for 12 h) [125]. It inhibited cell proliferation and promoted cell death during chronic exposure to an elevated temperature within the physiological febrile range (e.g. 40°C for 12 h). HSF2 was also found to modulate the constitutive expression of HSPs positively or negatively in mouse blastocysts, and its deficiency promoted cell death after a mild and long heat shock [126]. Moreover, in chicken DT40 cells, HSF2 deficiency blocked cell proliferation and induced cell death during chronic exposure to a mildly high temperature (e.g. 43°C for 72 h). In contrast to ubiquitously expressed HSF2, HSF4 is highly expressed in the mouse lens, but is not involved in the induced expression of HSPs. Interestingly, heat-inducible *non-HSP* genes are included among HSF4 target genes and HSF4 deficiency was shown to block the heat-induced expression of a subset of these genes, at least through a reduction in HSF1 binding, in the lenses of a 2-day-old mouse [66]. The overexpression of HSF4 may positively or negatively affect the induced expression of HSPs in lens epithelial cells and protect cells from exposure to proteotoxic stress inducers [127]. Of note, the single essential HSF1 in the yeast *Saccharomyces cerevisiae* was substituted with human HSFs and their functions under heat shock conditions were analysed. The findings obtained revealed that human HSF4 and a constitutively trimerized HSF1, but not HSF2, induced *SSA3* (*HSP70*) promoter-driven reporter gene expression, and yeast cells expressing one of the three factors acquired thermotolerance [128,129]. These findings strongly suggest that mammalian HSF4 contributes to heat resistance, at least in part by modulating HSF1 activity.

### (b) HSF-mediated pathways and their roles in heat resistance

Heat shock ultimately leads to apoptotic and necrotic cell death; however, the pathways of cell death and the factors primarily impaired by heat shock may be diverse. Cells in different states of metabolism and in different stages of differentiation may be induced to die by different mechanisms. Furthermore, the effects of heat shock on proteins and other molecules are dependent on temperature. For example, newly synthesized proteins are highly sensitive to heat shock and, thus, the blockade of protein synthesis inhibited HSF1 activation during the exposure of human cells to mild heat shock [130,131]. HSFs control the expression of a number of genes in addition to *HSP* genes, and the pathways mediated by the HSF target gene products (herein referred to as HSF-mediated pathways) may be involved in adaptation to heat shock as described below (figure 3).

**Figure 3.**
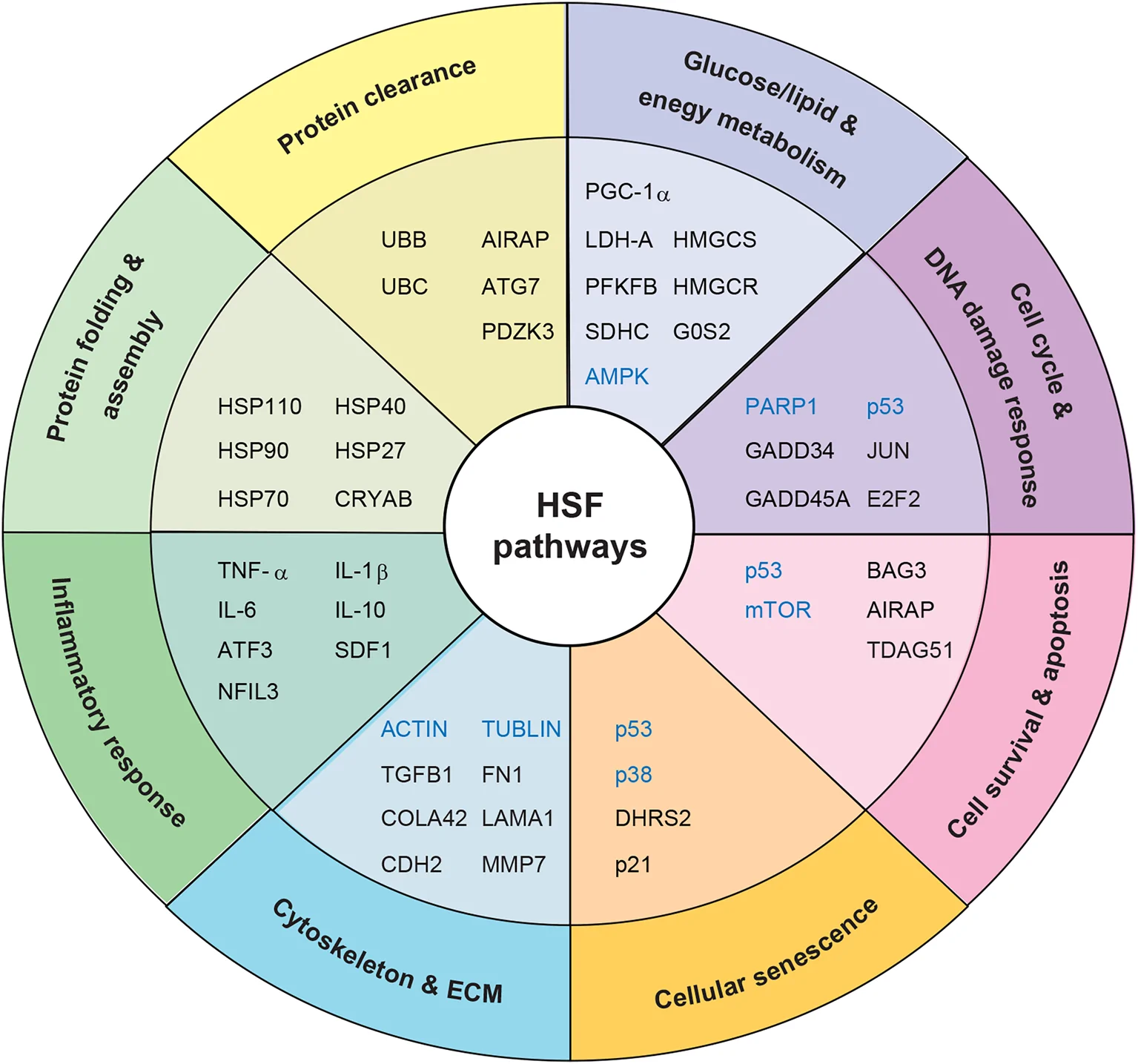
HSF-mediated pathways in vertebrates. The scheme illustrates the eight functionally classified categories of HSF-target gene products. Proteins activated or stabilized by HSFs are also included (blue). Organisms or cells in vertebrates acquire resistance to changing environmental temperatures primarily through the induction of chaperones. At the same time, the ability to resist changing temperatures may be modulated by many cellular functions mediated by a number of HSF-mediated pathways. ECM, extracellular matrix.

Mammalian cells contain more than 10  000 different proteins at concentrations ranging from 200 to 300 mg ml^–1^. The extremely high concentration of cellular proteins results in an excluded-volume effect (also known as macromolecular crowding), which promotes the aggregation of non-native proteins [132]. To maintain the proper conformations and physiological concentrations of numerous proteins, cells must preserve a balance between the synthesis, folding and clearance of individual proteins under a wide range of environmental and metabolic conditions [133,134]. An increase in temperature generally leads to proteostasis imbalance by causing protein misfolding and the formation of aggregates and amyloid fibrils, which are toxic to cells. HSPs, such as HSP110, HSP90, HSP70, HSP40 and small HSPs (HSP27 and CRYAB), cooperate to prevent protein misfolding and subsequent aggregate and amyloid formation under heat-shock conditions and facilitate their refolding during recovery from stress conditions [10,11]. At the same time, HSPs facilitate many cellular processes under heat-shock conditions, as discussed below. The expression and activity of protein degradation systems are also regulated in response to heat shock. The two polyubiquitin genes *UBB* and *UBC*, which contain HSEs in their promoters, were found to be upregulated in vertebrate cells by various stresses including heat shock [135,136]. Misfolded proteins induced by heat shock are rapidly polyubiquitinated and degraded by the 26S proteasome [137,138]. MEF cells deficient for UBC exhibited reduced cell proliferation with decreased steady-state ubiquitin levels, were unable to adequately increase ubiquitin levels, and were prone to death during heat shock [139]. In addition to the removal of misfolded proteins, ubiquitination is required for the disassembly of stress granules, translation and nucleocytoplasmic transport during recovery from heat shock [140]. In contrast to ubiquitin, components of the 26S proteasome are not upregulated upon heat shock. The 26S proteasome is activated in response to the accumulation of ubiquitinated proteins [141]. The proteasome-interacting protein AIRAP was previously shown to be upregulated following exposure to arsenite and heat shock and affected the biochemical properties of the 26S proteasome under stress conditions [142,143]. Autophagy is also activated in mammalian cells exposed to heat shock and contributes to the acquisition of thermotolerance [144,145]. HSF1 facilitates p62-mediated autophagosome formation in response to proteasome inhibition, at least through the upregulation of HSPs [146,147]. In addition, HSF1 directly binds to the promoter of *ATG7* gene and induces its expression during autophagy by a chemotherapeutic treatment, independent of heat shock [148]. Besides the major degradation systems, HSF1 upregulates the expression of PDZK—a scaffold of some proteins (e.g. polyglutamine proteins)—for degradation in MEF cells [37,149].

Glucose metabolism is regulated by HSF1 even in the absence of environmental stress. HSF1 deficiency has been shown to reduce glucose uptake and lactate dehydrogenase (LDH) activity in MEF cells [150]. In addition, HSF1 promotes glycolysis by upregulating the expression of 6-phosphofructo-2-kinase (PFKFB) in endometriosis cells [151]. Glycolysis is advantageous for the proliferation and survival of cells, specifically cancer cells [152]. The overexpression of ERBB2 (HER2) was shown to activate HSF1, which induced the expression of LDH-A by binding to its gene promoter and enhanced glycolysis and the proliferation of breast cancer cells [153]. HSF1 also directly regulates the expression of succinate dehydrogenase C (SDHC) to enhance mitochondrial oxidative phosphorylation in acute myeloid leukaemia stem cells [154]. Moreover, HSF1 controls lipid and energy metabolism. It directly regulates the expression of peroxisome proliferator-activated receptor γ coactivator-1α (PGC-1α), a transcriptional regulator of mitochondrial biogenesis and oxidative phosphorylation. The pharmacological activation of HSF1 promoted the expression of genes involved in mitochondrial biogenesis and thermogenesis, and enhanced energy expenditure through the upregulation of PGC1α in adipose and muscle tissues [155]. The HSF1-mediated induced expression of mitochondrial HSPs, such as mtHSP70, HSP60 and HSP10, has also been suggested to contribute to preserving mitochondrial function during the mitochondrial unfolded protein response [156,157]. AMP-activated protein kinase (AMPK) is an important regulator of energy homeostasis and an inhibitor of lipid synthesis. HSF1 was found to inhibit the activity of AMPK through a physical interaction [158]. HSF1 deficiency inactivates the lipogenic transcription factors SREBP1 and SREBP2 via AMPK-mediated phosphorylation. As a result, HSF1-null mice have a lower body fat mass owing to impaired lipogenesis and cholesterol synthesis [158,159]. HSF1 is also known to directly upregulate the expression of the cholesterol biosynthesis genes *HMGCS1* and *HMGCR* [160,161]. Cholesterol contributes to heat resistance by maintaining membrane fluidity [162]. In addition, G0S2, a negative regulator of lipolysis, is upregulated by HSF1 in human fibroblasts and partly contributes to resistance to daily temperature fluctuations [36].

Heat shock causes DNA damage, including single-strand DNA breaks (SSBs) and double-strand DNA breaks (DSBs), and interferes with DNA repair processes in a cell cycle-dependent manner [163]. For example, heat shock (e.g. 42°C or 45.5°C for 30 min) induces DSBs, which are marked by the phosphorylation of the histone H2AX (γH2AX) in human G1- and G2-phase cells, while heat shock arrests the progression of replication forks (also marked by γH2AX) in S-phase cells [164]. HSF1 plays roles in recovery from DNA damage caused by exposure to genotoxic reagents and ionizing radiation, and possibly by exposure to heat shock. Rat fibroblasts lacking HSF1 showed a reduced capacity to repair DSBs, which is associated with the diminished formation of foci containing the repair factor 53BP1 [165]. Poly (ADP-ribose) polymerase 1 (PARP1) is one of the first proteins recruited to DNA lesions, including DSBs. In response to DNA damage, it induces PARylation at DNA lesions and facilitates the recruitment of DNA repair factors, such as RAD51 and 53BP1. The HSF1–PARP13–PARP1 complex is widely distributed in the genome of human cells, and HSF1 facilitates the redistribution of PARP1 and the accumulation of γH2AX, RAD51 and 53BP1 in DNA lesions during DNA damage and efficiently promotes DSB repair [166]. HSF4 has also been shown to directly upregulate RAD51 expression in human lens epithelial cells, and its knock-down resulted in the accumulation of DNA damage in the lens of zebrafish [167]. On the other hand, the HSF1-mediated redistribution of PARP1 upregulated the expression of DNA damage-inducible genes, such as *GADD34* and *GADD45*, which are involved in the regulation of the cell cycle and apoptosis [166]. In addition, HSF1 directly upregulated the expression of the transcription factors JUN and E2F2, which direct the cell cycle-dependent expression of genes that induce the cell division process [168,169]. Furthermore, HSF1 was found to indirectly facilitate the expression of E2F target genes in human cancer cells as well as *C. elegans* [170,171].

HSF1 directly affects cell survival and apoptosis in a manner that is dependent on stress conditions. The transcription factor p53 may control the expression of genes involved in cell-cycle arrest, senescence and apoptosis in response to DNA damage stress. HSF1 co-occupies the *p21* promoters through physical interactions with p53, upregulates p21 expression and induces cell cycle arrest in MEF cells in response to genotoxic reagents [172]. Simultaneously, HSF1 enhances apoptosis, possibly by upregulating the proapoptotic protein Bax. Similarly, zebrafish HSF4 stabilizes p53 and is responsible for activating p53-mediated apoptosis signalling pathways during the terminal differentiation of lens fibre cells [173]. Conversely, human and zebrafish p53 were found to suppress HSF1 expression by directly binding to its gene promoter and promoted cell survival by inhibiting an excessive HSR under mild heat-shock conditions (e.g. 40°C for 16 h) [174]. p53 also suppresses HSF1 activity by upregulating the expression of the protein kinase CK2α’ and E3 ligase FBXW7, both of which are responsible for HSF1 degradation [175]. Bcl2-associated athanogene 3 (BAG3) is a member of the BAG family of co-chaperones that interact with HSP70 through the BAG domain, and is involved in many cellular processes including apoptosis, development, cytoskeleton organization, cell adhesion and autophagy [176]. BAG3 is transcriptionally induced by HSF1 [177] and protects cells from various types of stresses, including heat shock [176]. Another anti-apoptotic factor, AIRAP, also known as a proteasome regulator, is upregulated by HSF1 and inhibits apoptosis caused by proteasome inhibitor treatment through the stabilization of the cellular inhibitor of apoptosis 2 (cIAP2) [178]. The expression of the T-cell death-associated gene 51 (*TDAG51*), also known as *PHLDA1*, is induced by various stimuli, including heat, oxidative and inflammatory stimuli, and it promotes or inhibits apoptosis depending on the stress condition [179]. HSF1 induces the expression of TDAG51 and cell death during heat shock in mammalian cells, and TDAG51-mediated cell death is suppressed by HSPs [180]. The mTOR kinase plays a crucial role in promoting cell survival through the regulation of protein synthesis, cell growth and proliferation, and heat shock activates the JNK kinase that suppresses mTOR activity [181]. HSF1 inhibits JNK activation through physical interaction and preserves mTOR activity. This mechanism not only promotes resistance to proteotoxic stress but also an increase in cell and organ sizes [181]. HSF1 is also activated by mTOR-mediated phosphorylation at S326 [182].

Cell size is tightly controlled, and an increase in cell size promotes cellular senescence [183]. When human cells are chronically exposed to mild heat shock (e.g. 39°C for 7 days), these cells moderately increase their size without changing their cell cycle profiles in a manner that is dependent on HSF1 [184]. However, prolonged mild heat shock (e.g. 39°C for four weeks) leads to excessively large cells, which is associated with cellular senescence [184]. HSF1 was first shown to suppress DNA damage-induced senescence, in part by inhibiting the p38 kinase activity through unknown mechanisms [185]. Acute HSF1 depletion itself leads to the senescence of human immortalized fibroblasts, which is dependent on the activation of the p53-p21 pathway [186]. Regarding the underlying mechanism, HSF1 depletion upregulates the expression of DHRS2, a putative MDM2 inhibitor, which stabilizes and activates p53. HSF4 also suppresses cellular senescence in the lens. HSF4 upregulates the expression of p21 by binding to its gene promoter in mouse lens epithelial cells, and HSF4 deficiency promotes UVB-induced senescence in these cells [187]. Furthermore, mouse and zebrafish HSF4 deficiency has been shown to promote senescence in lens epithelial cells during development and ageing [187]. Interestingly, HSF4 deficiency inhibited spontaneous tumorigenesis in p53-depleted mice [188], similar to HSF1 deficiency [150,189]. It induced the senescence of MEF cells and cells in the normal and tumour tissues in association with the upregulation of the CDK inhibitors p21 and p27. The suppression of both normal cell proliferation (with or without heat shock) and tumour development by HSF deficiency may be related to cellular senescence in general.

Heat shock induces the rearrangement or collapse of major cytoskeletal networks: actin filaments, intermediate filaments and microtubules [190]. Among the hundreds of genes upregulated in MEF cells during heat shock, many of the early induced genes encode proteins with a biological function related to cytoskeletal structure and function in a manner that is independent of HSF1 [191]. Nevertheless, HSF1-mediated actin cytoskeletal integrity affects thermotolerance in *C. elegans*. HSF1 directly induces the expression of the calcium-binding protein PAT-10, thereby inhibiting the collapse of actin filaments by stabilizing them [192]. In human cancer cells, HSF2 has been shown to control the expression of GTPase effector proteins, including ROCK2 and WASF2, which regulate the assembly of actin filaments [193]. In addition, HSF1 facilitated the accurate assembly of spindle microtubules during the mitosis of human cells partially through the upregulated expression of HSP70 [194]. HSF1 may stabilize ciliary tubulins and facilitate their polymerization by the upregulation of HSP90 in the mouse cilia [195]. Many genes encoding the extracellular matrix (ECM) and cell adhesion proteins were revealed to be directly regulated by HSF1 in human cancer cells [196]. The expression of some ECM proteins (FN1, COLA42 and LAMB3) was induced by HSF1 in cancer cells in response to proteotoxic stress [197]. Importantly, HSF1 is also activated and upregulates the expression of secretory proteins, including TGF-β1, which increases ECM components and the chemokine SDF1, in cancer-associated fibroblasts that support cancer cells through the secretion of ECM proteins, chemokines, cytokines and growth factors [198]. Furthermore, HSF1 was found to be activated in stromal fibroblasts during the early stages of inflammation and induced ECM remodelling, which involves the upregulation of genes encoding matrix proteins (FN1 and LAMA1) and matrix remodelling enzymes (MMP7 and MMP9), thereby supporting the development of colon cancer [199]. Interestingly, HSF2 was shown to modulate the TGF-β1-mediated ECM remodelling by regulating the expression of ECM components in cancer cells [200]. HSF2 also upregulates the expression of a set of genes coding for cadherin superfamily proteins including CDH2 and CDH6, which mediate cell–cell adhesion, and this mechanism protects cells from chronic exposure to proteotoxic stress induced by proteasome inhibition [201].

The febrile response is a complex physiological reaction to disease, including a cytokine-mediated increase in body temperature and the activation of inflammatory systems. Fever or heat stress has beneficial roles in disease prognosis, in part by suppressing the expression of inflammatory cytokines [202]. HSF1 generally suppresses the production of pyrogenic and inflammatory cytokines, thereby preventing vertebrate animals from chronic exposure to febrile-range thermal stress and inflammation. HSF1 deficiency was found to increase mortality and exaggerate TNF-α production during lipopolysaccharide (LPS)-induced endotoxaemia in mice [203]. HSF1 suppressed TNF-α expression by binding to its gene promoter [204] and the expression of IL-1β through a physical interaction with its activator NF-IL6 [205]. The *IL-6* promoter is one of the promoters that are heavily occupied by HSF1 in MEF cells, irrespective of heat shock [31,206]. HSF1 opens the chromatin structure of the *IL-6* promoter to facilitate the binding of an activator and a repressor [207,208]. HSF1 directly induces the expression of the repressor ATF3 at elevated temperatures, and the LPS-induced febrile response is augmented in mice deficient for ATF3 as well as HSF1 [209]. Furthermore, HSF1 enhances the expression of genes adjacent to the HSF1 condensates around the *satellite III* DNA regions (known as nuclear stress bodies), including the transcription suppressor NFIL3. NFIL3 has been shown to strongly suppress inflammatory cytokines (TNF-α and IL-1β) in order to restrain the inflammatory response in human macrophages [210]. Moreover, the anti-inflammatory cytokine IL-10 is upregulated by HSF1 in mouse macrophages [211].

## 5. Concluding remarks

The HSR is a universal adaptive mechanism in all living organisms and is regulated by HSF family members in vertebrate animals, which experienced at least two rounds of WGD in their evolutionary history. Obviously, *HSF* genes have undergone functional diversification during evolution. Variations in their homeothermic capacity may be one of the major driving forces for the evolution of vertebrate *HSF* genes. Animals acquire resistance to changing environmental temperatures primarily through the induction of HSPs, which is mainly regulated by HSF1 and HSF3. It is just as important for adaptation to heat stress to modulate many cellular functions controlled differently by HSF family members. These HSF-mediated pathways play important roles not only in heat resistance, but also more generally in health and disease.

## Supplementary Material



## Data Availability

This article has no additional data. Supplementary material is available online [212].

## Theme

One contribution of 20 to a Theo Murphy meeting issue ‘ProteostaSys: a systems view of proteostasis’.
