# Supplementary material for: Adaptation to heat stress by diversification of the vertebrate heat shock transcription factor family
Source: Philos Trans R Soc Lond B Biol Sci. 2026 Oct 1;381(1960):20250274. doi: 10.1098/rstb.2025.0274 (PMC13628046; doi:10.1098/rstb.2025.0274)
Supplement: Supplementary file 1 [file rstb.2025.0274.zip › rstb.2025.0274/rstb20250274_si_001.doc]

**Supplementary Material**

**Adaptation to heat stress by diversification of the vertebrate heat shock transcription factor family**

**Ryosuke Takii, Mitsuaki Fujimoto, and Akira Nakai**

Department of Biochemistry and Molecular Biology, Yamaguchi University School of Medicine, Minami-Kogushi 1-1-1, Ube 755-8505, Japan

Supplementary Figure S1

Supplementary Figure S2

Supplementary Figure S3

Supplementary Figure S4

Supplementary references

**Supplementary Figure S1.** Phylogenetic trees of vertebrate HSF superfamily members.

(**A**) The phylogenetic tree was generated in MAFFT software version 7 [209] for members of vertebrate HSF family (except fish HSFs) and human HSF-related gene products (HSF5, HSFX1, and HSFY1). The unrooted tree was drawn using the NJ/UPGMA method. Numerals represent bootstrap values (1000 bootstrap replicates were performed). The bar represents 0.1 substitutions per site. HSF1, HSF2, HSF3, and HSF4 orthologs are indicated in different colors. The tree was constructed using amino acid sequences in human (*Homo sapiens*, NP_005517, NP_004497, NP_001361604, NP_001073908, NP_057237, NP_001412327), mouse (*Mus musculus*, NP_001318083, NP_032323, NP_001297683, NP_001242971), guinea pig (*Cavia porcellus*, XP_005000946, XP_012998949, XP_063101625, XP_003472079), squirrel (*Ictidomys tridecemlineatus*, XP_013211818, XP_021576053, XP_040144495, XP_005318355), armadillo (*Dasypus novemcinctus*, XP_004472077, XP_058163325, XP_058147078, XP_012381569), Tasmanian devil (*Sarcophilus harrisii*, XP_031803248, XP_031820197, XP_031800904, XP_031806165), koala (*Phascolarctos cinereus*, XP_020850493, XP_020842622, XP_020822907, XP_020845041), echidna (*Tachyglossus aculeatus*, XP_038616553, XP_038626061, XP_038603906), platypus (*Ornithorhynchus anatinus*, XP_028919528, XP_028912825, XP_028923365), cuckoo (*Cuculus canorus*, XP_053913726. XP_053919557, XP_053931782, XP_053935060), zebra finch (*Taeniopygia guttata*, XP_041570061.1, XP_012428115, XP_030128356, XP_030138332), mallard (*Anas platyrhynchos*, XP_027308641.1, XP_038032172, LC806256, XP_005025788), chicken (*Gallus gallus*, NP_001292185, NP_001161236, NP_001291970.1, NP_001165845), turtle (*Mauremys reevesii*, XP_039381390, XP_039385105, XP_039345502), alligator (*Alligator mississippiensis*, XP_014465302, XP_006273346, XP_014462741, XP_059569726), lizard (*Anolis sagrei*, XP_060632009, XP_060609249, XP_067327599, XP_067326983), and frog (*Xenopus tropicalis*, XP_031759585, XP_002937262, NP_001039053, XP_002937220).

(**B**) The phylogenetic tree was generated for members of the fish and human HSF families. HSF1, HSF2, and HSF4 orthologs are indicated in different colors. The tree was constructed using amino acid sequences of HSFs in Japanese pufferfish (*Takifugu rubripes*, XP_011607531, XP_029687955), southern bluefin tuna (*Thunnus maccoyii*, XP_042292492, XP_042246221, XP_042268223), medaka (*Oryzias laptipes*, XP_023815376, XP_011489975, XP_011474681), rainbow trout (*Oncorhynchus mykiss*, NP_001118220, NP_001118221, NP_001117849, XP_021456071), king salmon (*Chinook salmon*, XP_024296632, XP_024251169, XP_024275793, XP_024233754), zebra fish (*Danio rerio*, NP_001300665, NP_571942, NP_001315137), and whale shark (*Rhincodon typus*, XP_048473159, XP_020372566, XP_048461803).

**Supplementary Figure S2.** Diagrammatic representation of vertebrate orthologs of HSF1, HSF2, HSF3, and HSF4.

Percent identity of the amino acid sequences in domains or regions was estimated by comparing them with those of human HSF1, HSF2 and HSF4 or platypus HSF3. The numbers of amino acids of each HSF are shown. DBD, DNA-binding domain; HR, hydrophobic heptad repeat; DHR, downstream of HR-C; Region X, a region between the HR-A/B and HR-C; Region Y, a C-terminal region downstream of the DHR [15]. The red boxes in placental orthologs of HSF3 indicate HR-C-like domains, in which hydrophobic amino acids are not well conserved [59]. The transcriptional activation domains (ADs) and repression domains (RDs) were elucidated by a GAL4-driven luciferase reporter assay. The ADs in human HSF1 (a.a. 372-431) [36], HSF2 (a.a. 282-386 and a.a. 472-536) [72], and HSF4 (a.a. 296-395) [34] and platypus HSF3 [60] are indicated as red bars. The RDs in human HSF2 (a.a. 199-238 and a.a. 389-445) [72] and HSF4 (a.a. 200-295) [34] are indicated as blue bars. The regulatory domain (Regulatory D., a.a. 221-310) in the X region of human HSF1 is indicated as a green bar [36].

**Supplementary Figure S3.** Alignments of amino acid sequences for vertebrate HSF1 and HSF3 proteins.

(A) Alignments of amino acid sequences in the HSF1 regions surrounding major phosphorylation (S or T) and acetylation (K) sites are shown. Modifications positively or negatively affecting transcriptional activity are indicated as red and blue circles, respectively [35]. S320 and T529 phosphorylation sites (indicated by boxes) are conserved only in mammals or placentals, whereas other modification sites are conserved in most vertebrate species. The activation domain (AD) in human HSF1 is located in the C-terminal domain containing the HR-C, DHR, and Y region [36].

(B) Alignments of amino acid sequences in the HSF3 regions surrounding the HR-C and DHR and its C-terminal regions are shown. The activation domain (AD) in platypus HSF3 is located in the C-terminal domain containing the HR-C, DHR, and Y region [60], whereas that in chicken HSF3 is located in the upstream of the HR-C [61]. Classes and subclasses of vertebrate species are indicated on the right.

**Supplementary Figure S4.** Diagrammatic representation of fish orthologs of HSF1, HSF2, and HSF4.

Percent identity of the amino acid sequences in domains or regions was estimated by comparing them with those of human HSF1, HSF2 and HSF4 (at the bottom of each panel). The numbers of amino acids of each HSF are shown. DBD, DNA-binding domain; HR, hydrophobic heptad repeat; DHR, downstream of HR-C; Region X, a region between the HR-A/B and HR-C domains; Region Y, a C-terminal region downstream of the DHR domain [15].

**Supplementary references**

212. Mackowiak PA. 1998 Concepts of fever. *Arch Intern Med.* **158**, 1870-1881. (doi: 10.1001/archinte.158.17.1870)

213. Yousef H, Ramezanpour Ahangar E, Varacallo MA. 2023 Physiology, Thermal Regulation. *In: StatPearls [Internet]. Treasure Island (FL): StatPearls Publishing; 2025 Jan–.* (PMID: 29763018)

214. Abreu-Vieira G, Xiao C, Gavrilova O, Reitman ML. 2015 Integration of body temperature into the analysis of energy expenditure in the mouse. *Mol Metab.* **4**, 461-70. (doi: 10.1016/j.molmet.2015.03.001)

215. Akita M, Ishii K, Kuwahara M, Tsubone H. 2001 The daily pattern of heart rate, body temperature, and locomotor activity in guinea pigs. *Exp Anim*. **50**, 409-15. (doi: 10.1538/expanim.50.409)

216. Roberto R. 1996 The Body Temperature Rhythm of the Thirteen-Lined Ground Squirrel, Spermophilus tridecemlineatus. *Physiological Zoology* **69**, 270-275. (https://www.jstor.org/stable/30164183)

217. The University of Chicago Press. Sponsored by the Division of Comparative Physiology and Biochemistry, Society for Integrative and Comparative Biology 1996 *Physiological Zoology* **69**. (https://www.jstor.org/stable/i30164179)

218. Kjell J. 1961 Temperature Regulation in the Nine-Banded Armadillo (Dasypus novemcinctus mexicanus) *Physiological Zoology*, **34**, 126 -144. (https://www.jstor.org/stable/30152689)

219. Jones ME, Grigg GC, Beard LA. 1997 Body temperatures and activity patterns of Tasmanian devils (Sarcophilus harrisii) and eastern quolls (Dasyurus viverrinus) through a subalpine winter. *Physiol Zool*. **70**, 53-60. (doi: 10.1086/639541)

220. Mella VSA, Cooper CE, Karr M, Krockenberger A, Madani G, Webb EB, Krockenberger MB. 2024 Hot climate, hot koalas: the role of weather, behaviour and disease on thermoregulation. *Conserv Physiol*. **12**, coae032. (doi: 10.1093/conphys/coae032)

221. Schmidt-Nielsen K, et al. 1966 Temperature regulation in the echidna (Tachyglossus aculeatus). *J Cell Physiol.* PMID: 5937018 (doi: 10.1002/jcp.1040670108)

222. Smyth DM. 1973 Temperature regulation in the platypus, *Ornithorhynchus anatinus* (Shaw). *Comp Biochem Physiol* **45**, 705-715. PMID: 4147924. (doi: 10.1016/0300-9629(73)90074-1)

223. Ando S. 1995 Fluctuation of body temperature and cuckoo brood parasitism. *Ecol. Res.* **10**, 321-325.

(https://doi.org/10.1007/BF02347858)

(https://link.springer.com/content/pdf/10.1007/BF02347858.pdf)

224. Langman, Vaughan A. 1973 A Radio-biotelemetry System for Monitoring Body Temperature and Activity Levels in the Zebra Finch. *The Auk* *90*, 375-383.

(https://digitalcommons.usf.edu/auk/vol90/iss2/14)

225. Marais M, Gugushe N, Maloney SK, Gray DA. 2011 Body temperature responses of Pekin ducks (Anas platyrhynchos domesticus) exposed to different pathogens. *Poult Sci.* **90**, 1234-1238. (doi: 10.3382/ps.2011-01389)

226. Aluwong T, Sumanu VO, Ayo JO, Ocheja BO, Zakari FO, Minka NS. 2017 Daily rhythms of cloacal temperature in broiler chickens of different age groups administered with zinc gluconate and probiotic during the hot-dry season. *Physiol Rep.* **5**, e13314. (doi: 10.14814/phy2.13314)

227. Xu W, Dang W, Geng J, Lu HL. 2015 Thermal preference, thermal resistance, and metabolic rate of juvenile Chinese pond turtles Mauremys reevesii acclimated to different temperatures. *J. Therm. Biol*. **53**, 119-24. (doi: 10.1016/j.jtherbio.2015.09.003)

228. Rogerson G, Bock S, Loera Y, Parrott B, Mulley JF. 2023 Incubation temperature alters stripe formation and head colouration in American alligator hatchlings and is unaffected by estradiol-induced sex reversal. *J. Exp. Biol.* **226**, jeb245219. (doi: 10.1242/jeb.245219)

229. Rej JE, Gunderson AR. 2025 High temperatures amplify aggressiveness of an invasive lizard toward a native congener. *J. Therm. Biol.* **133**, 104257. (doi: 10.1016/j.jtherbio.2025)

230. Tuckett QM et al. 2024 Thermal tolerance for the tropical clawed frog, Xenopus tropicalis with comments on comparative methods for amphibian studies. *J. Therm. Biol*. **123,** 103890. (doi: 10.1016/j.jtherbio.2024.103890)
